# Practical advice on variable selection and reporting using Akaike information criterion

**DOI:** 10.1098/rspb.2023.1261

**Published:** 2023-09-27

**Authors:** Chris Sutherland, Darragh Hare, Paul J. Johnson, Daniel W. Linden, Robert A. Montgomery, Egil Droge

**Affiliations:** ^1^ Centre for Research into Ecological and Environmental Modelling, University of St Andrews, St Andrews, UK; ^2^ Wildlife Conservation Research Unit, Department of Biology, University of Oxford, Oxford, UK; ^3^ Department of Natural Resources and the Environment, Cornell University, Ithaca, NY, USA; ^4^ Northeast Fisheries Science Center, NOAA National Marine Fisheries Service, Woods Hole, MA, USA; ^5^ Department of Biology, University of Oxford, Oxford, UK; ^6^ Zambian Carnivore Programme, Mfuwe, Zambia

**Keywords:** information criterion, ecology, model selection, *p*-value, variable selection

## Abstract

The various debates around model selection paradigms are important, but in lieu of a consensus, there is a demonstrable need for a deeper appreciation of existing approaches, at least among the end-users of statistics and model selection tools. In the ecological literature, the Akaike information criterion (AIC) dominates model selection practices, and while it is a relatively straightforward concept, there exists what we perceive to be some common misunderstandings around its application. Two specific questions arise with surprising regularity among colleagues and students when interpreting and reporting AIC model tables. The first is related to the issue of ‘pretending’ variables, and specifically a muddled understanding of what this means. The second is related to *p*-values and what constitutes statistical support when using AIC. There exists a wealth of technical literature describing AIC and the relationship between *p*-values and AIC differences. Here, we complement this technical treatment and use simulation to develop some intuition around these important concepts. In doing so we aim to promote better statistical practices when it comes to using, interpreting and reporting models selected when using AIC.

## 1. Motivation

The debates around the use of *p*-values to identify ‘significant’ effects [1,2], Akaike information criterion (AIC) for selecting among models [3,4] and optimal model selection strategies [5] are lively, and in some cases divisive. In lieu of a consensus on these ongoing debates, we avoid the inherent theoretical and philosophical arguments and focus instead on the need for practical approaches and a deeper appreciation by end-users of how to interpret them and when to apply them [6]. Specifically, we address two questions that we encounter with surprising regularity in interactions with colleagues and students that are often prompted by seemingly misguided comments received during the peer-review process. These questions are:

1. What are ‘pretending’ parameters and how do they influence AIC model ranking?
2. Why is the *p*-value of an effect in the AIC-top model not always ‘significant’?

A variety of information criteria with different properties can be used to rank models, including the ‘deviance’ (DIC), ‘Bayesian’ (BIC) and ‘widely applicable’ (WAIC) information criteria [7–9]. Here, we focus on AIC as it currently dominates the ecological literature [10], although we suggest its properties are not always carefully considered when applied [7]. We acknowledge that there is a wealth of published information on multi-model inference and the use of AIC (e.g. [11]). We also acknowledge important contributions by Arnold [12] and Leroux [13] who succinctly and accessibly highlight the issue of uninformative parameters using ecological examples and provide guidance on how to identify uninformative parameters. Despite this, however, we often encounter many users from a wide range of backgrounds with an apparently muddled understanding of what it means for parameters to be ‘uninformative’, how to handle such parameters, and why this matters. Similarly, how model rankings emerge, and how to interpret models and the covariate effects included in them, are a continuing source of confusion. Here we use the valuable tool of simulation to provide a cognitive device to increase clarity and intuition around model and parameter selection using AIC [6].

For clarity, and to introduce some terminology, AIC is calculated as

AIC=2k−2ℓ,

where *k* is the number of parameters in the model and $\ell =\ln(\hat{L})$ is the log-likelihood of the data under the model. Simply put, AIC has two components: −2ℓ is the deviance, a measure of model fit that is a function of the likelihood of the model given a set of parameter values, and 2*k* is twice the number of parameters in the model and is, hence, a measure of model complexity. AIC, therefore, achieves parsimony via a fit-complexity trade-off and is used as a relative measure to compare and rank several competing models fit to the same data, where the model with the lowest AIC is considered the best [11].

## 2. On pretending

When using AIC to compare two models that are identical except for a single term, there are two possible outcomes. Take the following for example:

$$y_{i}=\beta_{0}+\varepsilon_{i}$$

and

$$y_{i}=\beta_{0}+\beta_{1}X_{i1}+\varepsilon_{i}.$$

We use *β* to denote regression parameters, *X* are covariates and ε are observation-specific residuals which are, in the case of this linear model that assumes Gaussian errors, normally distributed,

$$\varepsilon_{i}∼\text{Normal}(0,\sigma^{2}).$$

The first of these models is commonly referred to as the ‘null’ or ‘intercept-only’ model and has *k* = 2 parameters, one parameter for the intercept, *β*_0_, and one parameter for the residual variance, *σ*. The second model includes one additional covariate, and hence one additional parameter, *β*_1_, which is estimated effect of a unit change in the covariate on the response. The second model, therefore, has *k* = 3 parameters, the two regression parameters and the residual variance. We note that there is some inconsistency in the literature and between software in whether the residual variance parameter is formally included as part of the parameter count.

If the additional covariate (*X*_1_) is statistically important, i.e. it explains enough variation in the response to warrant being considered when drawing inference, the model will generally have a *lower* AIC despite the two-unit penalty for the one additional parameter, and thus be ranked higher. If, on the other hand, the covariate has no association at all with the response, the likelihood will increase given that adding parameters will improve the fit of the model by explaining some of the randomness, but the penalty of the additional parameter will mean that the model will generally have a *higher* AIC. The latter is referred to as a ‘pretending’ variable [11], and the result is a model including a completely uninformative term will appear competitive as it will be within 2 AIC units of the model without that parameter [12,14]. In these cases, strategies are provided to address these uninformative terms [12,13], all of which recommend that models containing such terms should not be blindly accepted as being statistically supported.

One approach to better understanding this issue is through simulation. Here we simulate a response variable, *y*, that is positively associated with one explanatory variable, *X*_1_, and that has no association with a second explanatory variable, *X*_2_. The model we simulate from is

$$y_{i}=\beta_{0}+\beta_{1}X_{1i}+\beta_{2}X_{2i}+\varepsilon_{i}$$

and

$$\varepsilon_{i}∼\mathrm{Normal}(0,\sigma^{2}).$$

**Figure 1.**
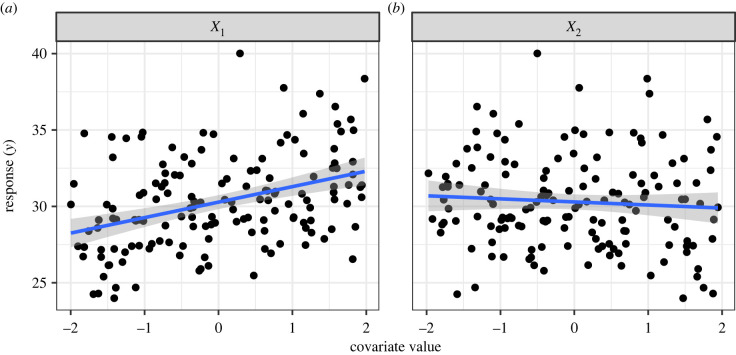
Simulated relationships between the response variable *y* and two covariates. The effect of *X*_1_ is positive (*β*_1_ = 1), and *X*_2_ has no effect (*β*_2_ = 0). Black points are simulated data points, the blue line is the estimated relationship from a univariate linear model with the focal covariate as the predictor. The shaded areas are corresponding 95% CIs around the expected relationship. Note that although the simulated effect size of *X*_2_ was 0, the regression slope is not perfectly horizontal; hence *X*_2_ will often explain a small amount of variation in the data simply by chance.

For this illustration we simulate 150 observations of *y* under this model using an intercept of *β*_0_ = 30, a positive *X*_1_ effect, *β*_1_ = 1, and a standard deviation of the residual error of *σ* = 3. We ensure that *X*_2_ has no influence by setting *β*_2_ = 0. The covariates *X*_1_ and *X*_2_ are uniform [ − 2, 2] random values resembling standardized covariates (figure 1). We note here that the code to conduct all the simulations in this paper is available as a supplement [15].

With a single response variable and two covariates, and not considering any interactions, we can fit the following four models and rank them using AIC in conditions where truth is known. They are the ‘full’ or ‘global’ model (*m*_3_), and all nested models including the ‘null’ or ‘intercept-only’ model (*m*_0_),

$$\begin{array}{rl} m_{0}:\;\;y_{i} & =\beta_{0}+\varepsilon_{i}\\ m_{1}:\;\;y_{i} & =\beta_{0}+\beta_{1}X_{1}+\varepsilon_{i}\\ m_{2}:\;\;y_{i} & =\beta_{0}+\beta_{2}X_{2}+\varepsilon_{i}\\ \mathrm{and}\;m_{3}:\;\;y_{i} & =\beta_{0}+\beta_{1}X_{1}+\beta_{2}X_{2}+\varepsilon_{i}. \end{array}$$

Here, *m*_0_ is the intercept-only, or null, model, *m*_1_ is the data-generating model and models *m*_2_ and *m*_3_ are models containing the uninformative parameter as either the only covariate or a second covariate, respectively.

Simulation furnishes us with perfect knowledge so we can fit models to simulated data and explore model rankings in the context of the known parameter redundancy. Looking at the AIC model rankings (table 1), the model with an uninformative parameter, *m*_3_, increases the likelihood relative to *m*_1_, the data-generating model and is, therefore, a better fit, and it has an AIC very close to the top model (ΔAIC = 1.95), suggesting some degree of support [11]. The improvement in terms of likelihood is expected: adding parameters improves model fit, but the fact the model performs similarly when ranked by AIC is exactly the *2 AIC problem* described by Arnold [12]. Indeed, AIC penalizes model complexity by 2 AIC units per additional term, so when we recognize that the model with the uninformative term is the AIC-top model plus a term that offers no meaningful additional information about the response and instead soaks up some of the randomness, blindly ranking models using AIC alone allows uninformative parameters to infiltrate the conversation about variable importance. This is clearly shown here where we have simulated a response variable with no relationship with *X*_2_ (*β*_2_ = 0), and yet the model that includes *X*_2_ has a ΔAIC of 1.95 (table 1).

**Table 1.** Model selection table from a single iterated simulation where models are ranked by AIC from lowest (AIC-top) to highest. The table includes the number of parameters, *K*; both AIC and ΔAIC which is AIC minus the lowest AIC and the negative log-likelihood, L.

model	description	*K*	L	AIC	ΔAIC
*m*_1_	data-generating model	3	−368.05	742.27	0.00
*m*_3_	data-generating + uninformative term	4	−367.97	744.22	1.95
*m*_0_	intercept-only model	2	−379.83	763.73	21.46
*m*_2_	uninformative term only	3	−379.43	765.03	22.76

In the example above, we simulated a single dataset from the model, and using a different seed value in the supplementary code could produce a situation with different ΔAIC values. Thus, while this single iteration offers a nice demonstration, it is instructive to simulate many more iterations to examine the expected pattern from the hypothetical data and model formulation. Thus, we stochastically simulate 1000 datasets under the same data-generating model and record the difference in AIC between models that include both the informative and uninformative terms and the AIC-top model (i.e. $\Delta {\mathrm{AIC}}_{m_{3}}$), noting that if $\Delta {\mathrm{AIC}}_{m_{3}}>0$ then *m*_3_ is ranked below the top model, and if $\Delta {\mathrm{AIC}}_{m_{3}}=0$ *m*_3_ is the top model. We also compute the difference in AIC between the model that includes the uninformative term only, *m*_2_, and the null model, *m*_0_, which is again, a comparison of two models that differ only by the single uninformative term ($\Delta {\mathrm{AIC}}_{m_{2}-m_{0}}$).

**Figure 2.**
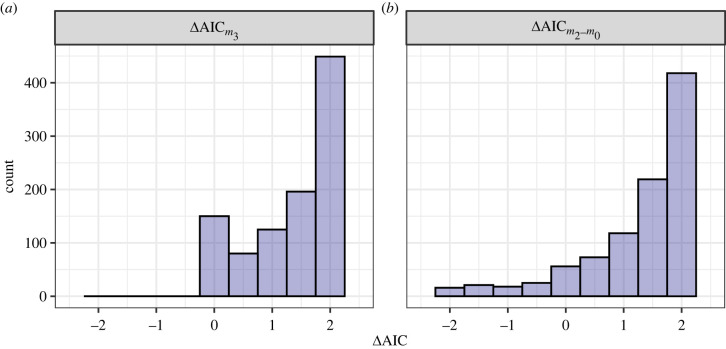
Comparisons of AIC differences between two indicative pairs of models. The first comparison (*a*) is the difference between *m*_3_, the data-generating model with the uninformative parameter included, and the top model. This histogram has a mass at 0 representing simulations where *m*_3_ was the top model. The second comparison is the ΔAIC between the null model (*m*_0_) and the model with only the uninformative term (*m*_2_). To facilitate comparison, we truncated the *x*-axis of (*b*) at −2 which removes the 4% of the data lying between −2 and approximately −12.

First, considering $\Delta {\mathrm{AIC}}_{m_{3}}$ (figure 2*a*), we see that the difference in AIC is not always exactly 2 (range: 0–2.11), which is what we might have expected when adding a single uninformative term and incurring a 2 unit penalty. This variation reflects the stochastic data-generating process akin to sampling variation in real-world data collection. The same pattern emerges for $\Delta {\text{AIC}}_{m_{2}-m_{0}}$ (figure 2*b*). In most cases *m*_2_ has a higher AIC and is thus ranked lower than the null model. However, just by chance, *y* can be correlated with *X*_2_ which results in a relatively large reduction (improvement) in AIC; as much as $\Delta {\mathrm{AIC}}_{m_{2}-m_{0}}=-10.53$. As a result, the AIC differences have a much wider range for $\Delta {\mathrm{AIC}}_{m_{2}-m_{0}}$ (range: −10.53–2.08), although the majority of cases have $\Delta {\mathrm{AIC}}_{m_{2}-m_{0}}>0$.

Interestingly, and seemingly counter to the point we are making, there are instances where *m*_3_, the model featuring the uninformative term, has a *lower* AIC than the data-generating model, and is actually the top ranked model, shown by the truncation at 0 (figure 2*a*). This is the case in 12.8% of the simulations. The result is mirrored for $\Delta {\mathrm{AIC}}_{m_{2}-m_{0}}$: using AIC to rank models, the model with a single uninformative term is also ranked higher than the null model in 13.8% of the simulations. These are false positives (Type I errors) which we will return to in the next section.

So, through simulation, where the truth is known, we have demonstrated that uninformative terms can appear in models that receive some support when using established rules of thumb [14]. We need not rely exclusively on decoding AIC model tables to identify pretending variables, though. It follows, and hopefully now in an intuitive way, that the estimate of the effect size should be informative about whether a variable is pretending. Indeed, despite being included in a highly ranked model, the uninformative parameter in our simulated example has a large *p*-value suggesting a lack of statistical support (*p* = 0.69, table 2). The estimate of the effect of *X*_1_ is estimated without bias and, in contrast, has a *p*-value that suggests convincing evidence of an effect, i.e. as being *significant* (*p* ≪ 0.05, table 2). It would appear here that we are ‘mixing paradigms’ by using *p*-values to interpret AIC-based model selection [16], but in fact, there is an explicit link between AIC differences and *p*-values. Neatly, this often underappreciated relationship [1,12] holds the answer to the second common question: *Why is the* *p*-*value of an effect in the AIC-top model not always ‘significant’*?

**Table 2.** Model coefficient table from the AIC-top model selected from the single iterated simulation. Each row is a parameter in the model, and the columns are the maximum-likelihood estimate and the corresponding standard error (s.e.) *t*-statistic (*t*-value) and *p*-value (Pr(>|*t*|)).

	estimate	s.e.	*t*-value	Pr(>|*t*|)
(intercept)	30.28	0.23	130.48	0.00
*X*_1_	1.00	0.20	4.93	0.00
*X*_2_	−0.08	0.21	−0.40	0.69

## 3. On ‘significance’

Many will be familiar with the *likelihood ratio test* (LR test) and its use as a method to select between two models. Returning to the situation where we wish to compare two models that are identical except for a single term, say Model *A* and Model *B* that has one additional parameter, or our *m*_1_ versus *m*_3_ above, the LR test is applied by first computing a likelihood ratio statistic,

$$\lambda_{\mathrm{LR}}=-2(\ell_{A}-\ell_{B}),$$

which is −2 times the difference in the log-likelihoods of the two models. This statistic is assumed to be Chi-squared distributed with degrees of freedom being the difference in the number of parameters, which in this case is *q* = 1. If the test statistic is greater than some *a priori* defined critical value, then the null hypothesis, that there is no improvement with the added complexity, is rejected and the more complex Model *B* has support. The critical value is defined by the significance level, *α*, which in the vast majority of cases is set to 0.05, i.e. the *p*-value will be equal to or below 0.05 when the test statistic is equal to or greater than the critical value.

What is interesting, and often underappreciated, at least in practical applications of AIC-based model comparisons, is that comparing models using AIC is closely related to the likelihood ratio test. For example, based on the equation for AIC given above, the difference in AIC scores between two models, or the ΔAIC, can be rewritten in terms of *λ*_LR_,

$$\begin{array}{rl} \Delta {\mathrm{AIC}}_{B} & ={\mathrm{AIC}}_{B}-{\mathrm{AIC}}_{A}\\ & =(2k_{B}-2\ell_{B})-(2k_{A}-2\ell_{A})\\ & =2(k_{B}-k_{A})-2(\ell_{B}-\ell_{A})\\ & =2q-2(\ell_{A}-\ell_{B})\\ & =2q-\lambda_{\mathrm{LR}}. \end{array}$$

When *q* = 1, i.e. model *B* has one more parameter than Model *A*, then Model *B* ranks higher than Model *A* when ΔAIC < 2, or in other words, when *λ* is at least 2. Under the Chi-square distribution with one degree of freedom, this corresponds to a significance level of *p* = 0.157. This is generally true for large samples sizes (e.g. *n*/*k* > 40 see [12]), and a more thorough treatment of this equivalence can be found elsewhere (e.g. [1]). Nevertheless, model selection using AIC in this simple case can be seen as functionally equivalent to conducting a likelihood ratio test with a more liberal significance level of *p* = 0.157 rather than *p* = 0.05. This also implies that for a parameter to improve the AIC relative to a model with one fewer terms, it would require *p* < 0.157 and *not* the conventional *p* < 0.05.^1^ In contrast to the *uninformative* variables, i.e. those that are not important but appear in top performing models by association only, we refer to variables that appear in the top model but have a *p* > 0.05 as *confusing* variables. We use this term specifically to reflect a confusion that arises when mixing the *p*-value and AIC model selection paradigms [16].

**Figure 3.**
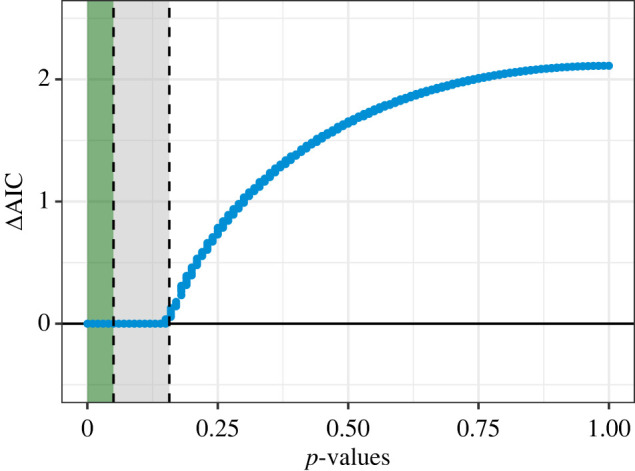
The relationship between $\Delta {\mathrm{AIC}}_{m_{3}-m_{1}}$, which is the difference between *m*_3_ and *m*_1_, and the *p*-value of *β*_2_. Vertical dashed lines are added at the conventional significance level of *p* = 0.05 (left), and at *p* = 0.157 (right), which is the threshold for obtaining a lower AIC than a nested model without the additional term. These thresholds define two relevant information zones: the green shaded is the zone of no confusion where AIC and *p*-values will select the same variable, and the grey area is the zone of confusion, where AIC will select variables that have *p*-values less than or equal to 0.157 but greater than 0.05. When $\Delta {\mathrm{AIC}}_{m_{3}-m_{1}}=0$ (the solid horizontal line), then *m*_3_ is the top ranked model based on AIC ranking.

This idea can be seen using the simulation results from the previous section. To do so, we use the comparison of the data-generating model (*m*_1_) and the data-generating model with a one additional term (*m*_3_), noting that even though the effect of the additional parameter was *β*_2_ = 0, there can be correlation between the response and *X*_2_ just by chance. Plotting the *p*-value of *β*_2_ against the difference in AIC between models *m*_3_ and *m*_1_, an almost deterministic relationship emerges (figure 3). The result clearly shows that *m*_3_, the model including the additional covariate *X*_2_, was always ranked as the top model, i.e. $\Delta {\mathrm{AIC}}_{m_{3}}=0$ when the *p*-value for *β*_2_ is less than or equal to 0.157, which is exactly as expected given the equivalence described above.

We can explore this behaviour further using a more complex data-generating model. Consider the following linear model with six covariates:

$$y_{i}=\beta_{0}+\beta_{1}X_{1i}+\beta_{2}X_{2i}+\cdots +\beta_{6}X_{6i}+\varepsilon_{i}$$

and

$$\varepsilon_{i}∼\mathrm{Normal}(0,\sigma^{2}).$$

**Figure 4.**
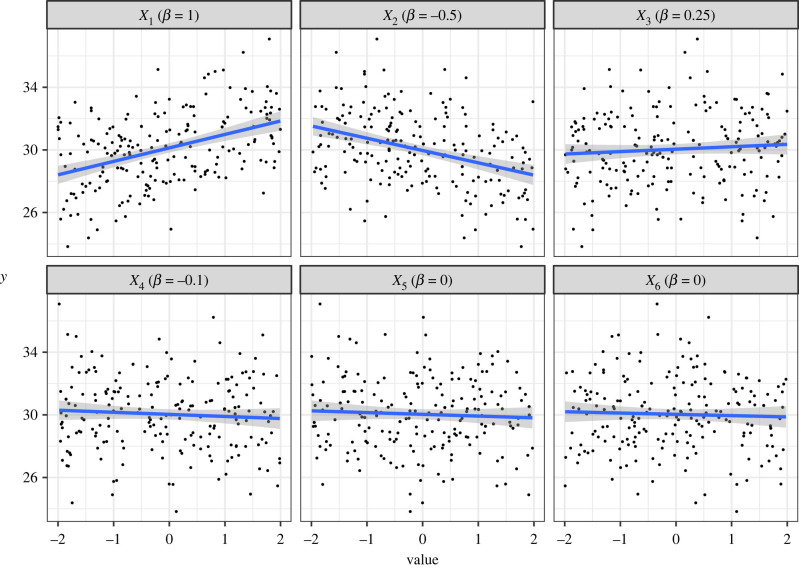
Simulated relationships between the response variable *y* and six covariates. The effects of *X*_1_ to *X*_6_ are *β* = (1, −0.5, 0.25, −0.1, 0, 0), respectively. Black points are simulated data points, the blue line is the estimated relationship from univariate linear model with the focal covariate as the single predictor, and the shaded areas are 95% CIs around the expected relationship.

As above, *β*_0_ is the intercept and *σ* is the standard deviation of the residual error. This time, however, we have six covariates, and therefore six regression coefficients, i.e. *β*_1_, …, *β*_6_. For this demonstration, we simulate a dataset of 200 observations under the model above using *β*_0_ = 30, *σ* = 3, and the values for *β*_1_, …, *β*_6_ are 1, −0.5, 0.25, −0.1, 0, 0, respectively. So, here, the first four covariates have non-zero effects that decrease in magnitude, while the effect of the last two covariates is set to 0 (figure 4).

Our aim here is to demonstrate that, based on the ideas presented above, it should come as no surprise that models that are selected based on having the lowest AIC values can, and *should* contain variables that have *p*-values that are greater than 0.05, but also that none of the variables featured in the AIC-top model will have a *p*-value greater than 0.157 (provided the null model is included in the model set). To do so, we fit the full model to one realization of simulated data, i.e. the model including all six covariates, and then use AIC to find the model with the lowest AIC, which we refer to as the ‘top model’. Estimates from the full model are shown in table 2 along with the coefficient *p*-values. We also report the standard 95% CI corresponding to the 0.05 significance level and the 85% CI that correspond approximately to the 0.157 significance level and indicate whether each effect is retained in the AIC-top model. Parameters with *p* < 0.157 are retained in the AIC-top model which would, if viewed through the lens of *α* = 0.05, seem counterintuitive at first glance, but when considered in the context of the equivalence described above, it is in fact expected (table 3).

**Table 3.** Coefficient estimates from the full model with corresponding *p*-values, 95% and 85% CIs, and whether or not the variable was selected in the top AIC model. The *p*-values are coloured as green if *p* < 0.05, orange if 0.05 < *p* < 0.157, and red if *p* > 0.157. The estimates, confidence intervals and *p*-values are slightly different for the reduced model selected by AIC, and full model tables from both models are provided in electronic supplementary material, tables S1 and S2.

		95% CI		85% CI			
covariate	estimate	lower	upper	lower	upper	*p*-values	AIC top
1	0.85	0.607	1.084	0.671	1.021	0.000	✓
2	−0.75	−0.997	−0.506	−0.931	−0.572	0.000	✓
3	0.22	−0.015	0.457	0.048	0.394	0.067	✓
4	−0.17	−0.401	0.064	−0.339	0.002	0.154	✓
5	−0.14	−0.388	0.106	−0.322	0.040	0.262	×
6	0.07	−0.185	0.329	−0.116	0.260	0.581	×

**Figure 5.**
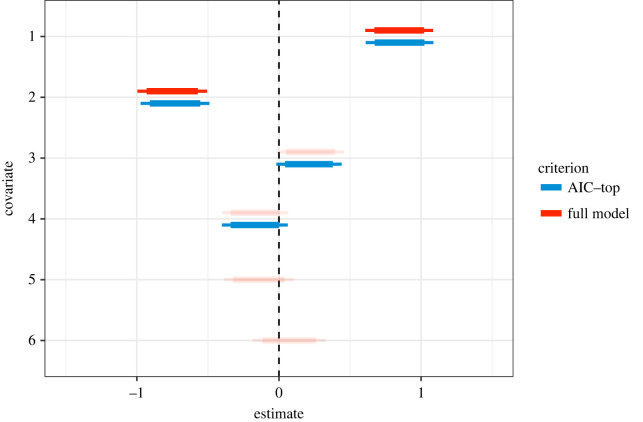
Coefficient plot where the thick and thin horizontal lines are the covariate-specific 85% and 95% CIs, respectively. Red lines show estimates under the full model where all six covariates are included in the model and, as such, coefficients for all covariates are estimated, and transparency is added to estimates with 95% CIs that overlap 0. Blue lines are the estimates obtained from the model with the lowest AIC; this model does not contain all covariates and therefore has missing estimates. The vertical line is at 0. The 85% CI is reported as it is consistent with how terms are selected under the AIC-based model selection criteria.

An interesting conundrum emerges here: if an AIC model selection approach is adopted, is a 95% CI the most appropriate interval to report in tables and figures? We believe that reporting 95% CIs often draws a reader’s (or reviewer’s) attention to whether these intervals overlap zero, and if they do, which they regularly do for covariates in model selected using AIC, whether the effect is ‘significant’ in the traditional *α* = 0.05 sense. The inevitable questioning of whether a term should be reported as being important despite having 95% intervals that overlap 0 is, we believe, a second source of confusion that arises from the underappreciation of the link between AIC-based model selection and *p*-values. This apparent misunderstanding of variable selection using AIC can be alleviated quite easily by first understanding the equivalence, but also by reporting either only the 85% interval, consistent with the model selection strategy, or the 85% interval along with the more commonly reported 95% interval, and in both cases providing an explicit statement about the 85% interval being consistent with how variables are selected when using AIC [12] (e.g. figure 5).

It is important to note that another strategy for interpretation of all parameters, not just confusing ones, is to focus on relative effect sizes. Doing so, parameters that suggest strong evidence for relationships can be highlighted, while those with weak evidence, which is typical of confusing parameters, are given less attention. This is illustrated in our example where, although models containing parameters 3 and 4 were supported by lower AIC values, the effect sizes were smaller than parameters 1 and 2, and there was a greater chance that the true effect size could have been the opposite direction of our point estimates. This nuance is particularly important for studies using observational data, where in any single study, many measured and unmeasured relationships are likely to be at play to varying degrees of importance [5].

## 4. Summary

We recognize that many common statistical practices are misleading, regardless of the technical accuracy with which they are executed and described [6]. Many of our ecology and conservation science colleagues and students who identify as end-users of statistical methods are either unaware of, or admit confusion about, the details of how variables are selected when using AIC to select models. This stems from a more general underappreciation of the explicit link between AIC differences and *p*-values. Motivated by what we perceive to be an avoidable misunderstanding about these practically important features of AIC model selection, we presented an unapologetically non-technical demonstration of these key ideas using simulation, with the aim of promoting better statistical practices when it comes to using, interpreting and reporting models selected when using AIC. Through this demonstration, we have attempted to develop some intuition around four important concepts that we hope go some way towards achieving these aims.

1. *Pretending variables/uninformative parameters*. Ranking nested models using AIC amounts to ranking by deviance with a complexity penalty related to the number of parameters. This means that models that are structurally similar to a ‘top’ model, but that contain covariates that explain very little or none of the variation in the data, can rank highly in model selection tables (table 1). These are what have been described as ‘pretending’ [11] or ‘uninformative’ [12,13] variables, and while we do not provide any novel recommendations for handling these cases, we provide clarity on how they manifest and encourage readers to adopt existing recommendations [12,13], but with a renewed appreciation for their relevance when interpreting model selection tables.
2. *AIC–p-value equivalence*. There is a close and explicit link between the likelihood ratio test and AIC differences (the ΔAIC). In terms of comparing models, the key difference amounts to the confidence threshold being used; specifically that, unlike the more commonly applied and accepted threshold of *p* < 0.05, model selection by AIC is functionally equivalent to defining a more liberal significance level of *p* < 0.157. This equivalence has been highlighted previously [1,3], but is probably more appreciated among statisticians than the majority of, for example, applied ecologists or conservation practitioners who regularly engage in statistical analyses that involve formally comparing among models. In contrast to the existing technical literature, we explore the equivalence through simulation, in an attempt to provide an accessible and intuitive appreciation of how these two apparently conflicting variable selection paradigms are closely linked.
3. *Confusion*. Unlike the uninformative parameter (see 1), we introduced a second type of parameter that we find to be a source of uncertainty—a *confusing* parameter. The confusion we are drawing attention to is the apparent ‘non-significance’ of a variable in the top model, which will be transparent if *p*-values are reported. The confusion arises from mixing model selection paradigms and incorrectly using *p* < 0.05 thresholds to interpret variable importance in AIC-top models [11]. We have shown that the difference in significance thresholds implied by the two approaches (see 2) means that, in contrast to the *α* = 0.05 viewpoint, parameters will appear in AIC-top models if they have a *p*-value below a more liberal threshold of *α* = 0.157. We also note that when reporting parameter estimates, an additional but related problem arises if 95% CIs for these confusing parameters as the intervals will span zero. This is regularly interpreted as lack of support for an effect, in line with the widespread tendency to dichotomize predictors as either ‘significant’ or ‘not significant’. Whether or not this is good practice, it is clear from the preceding exploration that 85% CIs are perhaps more appropriate to describe uncertainty for parameter estimates in models selected using AIC values. We note also that an examination of effect sizes (relative to other parameters) is crucial for the interpretation of confusing parameters while acknowledging that only additional data can clarify the relationships suggested by the model.
4. *Reporting*. By highlighting some of the key similarities and differences between two of the mainstream approaches available to end-users for selecting among models and identifying variable importance, we recommend that the statistical reporting should be consistent with the specific selection paradigm used. For example, because any confidence interval of a parameter estimate from most standard statistical models can be constructed from the standard error, we suggest reporting standard errors in addition to confidence intervals. Moreover, if confidence intervals are reported or plotted, we recommend being explicit about which intervals are reported and why, and ensuring that the intervals used reflect the model selection strategy used. For example, when using AIC for model selection, reporting either only the 85% interval, which is consistent with the model selection strategy, or both the 85% and the more commonly reported 95% interval, but in both cases unnecessary confusion about the use of so-called ‘unconventional’ intervals can be avoided by explicitly justifying why they are reported. Plotting multiple intervals (as we did in figure 5) can make clear how the results fit within the context of more than one inferential perspective, giving readers the option for multiple interpretations.

We conclude with one final consideration, which is that AIC is not always the optimal tool for a given modelling exercise [5]. AIC can tend to favour complex models due to its default prior model weighting, a fact that seems underappreciated [7] given how often the properties of AIC as a model selection criterion do not align with the objectives of a published study. AIC was popularized among ecologists in part as a method to address the potential structure of complex observational processes in hierarchical models of natural systems [11]. It is intended for making predictive inferences, not necessarily causal assertions. Our goal here is not to expound on the justifications for AIC in ecological applications, but instead we simply hope to enable better statistical practices when using this tool.

## Figures and Tables

**Figure 6.**
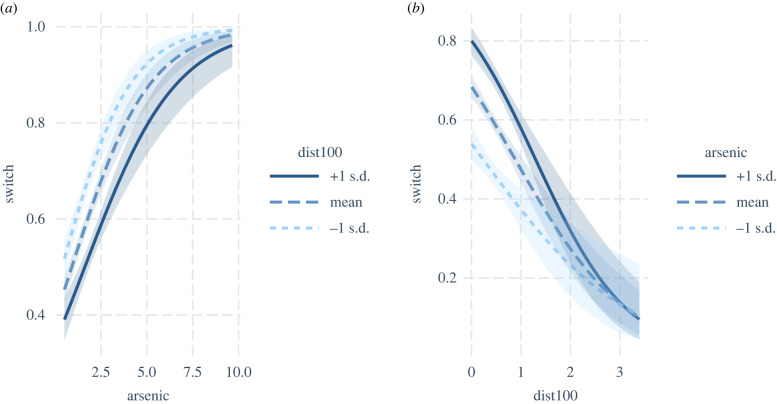
Predicted probability of switching wells as a function of distance to the nearest alternative (*a*) and arsenic levels (*b*). For each predictor, the predictions are made at the mean, and plus and minus 1 standard deviation of the other.

## Data Availability

All the code to produce the simulations, and associated figures and tables, are available via the following Open Science Framework project: https://doi.org/10.17605/OSF.IO/PTCB5 [15]. Supplementary material is available online [17].

## Footnotes

1 We take the liberty of omitting technical detail linking the *p*-value of the model comparison and the *p*-value of the additional model coefficient, but the current presentation works as a cognitive device.

## Appendix A. A brief illustrative example

We briefly discuss a textbook example that highlights some of the points we have addressed in the simulation exercise in the main text. This example is particularly interesting because the original application did not actually employ AIC. Gelman *et al.* [18] illustrate a ‘well-switching’ problem that uses logistic regression to estimate the probability that a household in Bangladesh would switch from a home drinking well to a nearby alternative based on the level of toxic arsenic contamination in the home well. The simple two-variable model finds that both arsenic level and distance to an alternative well are significant predictors (*p* ≪ 0.05). An interaction between arsenic and distance is added to the model (i.e. one term is added) with equivocal results, suggesting the interaction may not be needed.

In replicating the example (see code below), we note that parameter estimates using maximum likelihood (table 4) closely match the Bayesian posterior distributions (Gelman *et al.* [18]; section 14.2). The interaction term has a *p*-value of *p* = 0.08, making it *non-significant* at an alpha of 0.05, yet as expected, the additional term improves the AIC enough to make it the top model (table 5). In this example, the authors use different model selection criteria to conclude that the additional term does not change the predictive performance and is, therefore, unnecessary. However, it is still plausible for the interaction to be describing a true relationship (figure 6). Further interpretation could acknowledge the effect sizes as indicating weak evidence for the interaction.

**Table 4.** Coefficient table from the ‘well-switching’ model of Gelman *et al.* [18] showing the maximum-likelihood estimates, standard errors and associated *p*-values for the four parameters. Note that the distance × arsenic interaction term is not significant at the 5% level, but as seen in table 5, is in the top model when using AIC (i.e. it is a ‘confusing’ or ‘pretending’ variable).

parameter	estimate	s.e.	*p*-value
intercept	−0.148	0.118	0.20838
distance	−0.578	0.209	0.00579
arsenic	0.556	0.069	0.00000
distance × arsenic	−0.179	0.102	0.08040

**Table 5.** AIC model selection table comparing AIC scores for two formulations of the ‘well-switching’ model of Gelman *et al.* [18]: a model with a distance × arsenic interaction, and a model without the interaction term. Here, the model with the interaction term is preferred when using AIC, unlike in the original analysis.

model	AICc	no. parameters	AICc	AIC weight	log-likelihood
interaction	3935.64	5	0.00	0.63	−1963.81
no interaction	3936.68	4	1.00	0.37	−1965.33
